# Maintenance of skeletal muscle mass during FOLFIRINOX is a favorable prognostic factor in pancreatic cancer patients

**DOI:** 10.1186/s13104-021-05681-x

**Published:** 2021-07-15

**Authors:** Dong Woo Shin, Minseok Albert Kim, Jong-chan Lee, Jaihwan Kim, Jin-Hyeok Hwang

**Affiliations:** 1 Department of Internal Medicine, Seoul National University College of Medicine, Seoul National University Bundang Hospital, 82, Gumi-ro 173 beon-gil, Bundang-gu, Seongnam, Gyeonggi-do 463-707 South Korea; 2 Division of Gastroenterology, Department of Internal Medicine, Keimyung University School of Medicine, Keimyung University Dongsan Medical Center, Daegu, Republic of Korea; 3 Department of Internal Medicine, Seoul National University College of Medicine, Seoul National University Hospital, Seoul, South Korea

**Keywords:** Locally advanced pancreatic cancer, Skeletal muscle, Adipose tissue, Prognosis, FOLFIRINOX

## Abstract

**Objective:**

The study aimed to investigate the effect of body composition changes during chemotherapy on clinical outcomes in patients with pancreatic cancer.

**Results:**

In patients with locally advanced pancreatic cancer (LAPC), the cross-sectional area of skeletal muscle (SM) and adipose tissue (AT) at the level of third lumbar vertebra was measured. The SM and AT ratios indicated the changes during chemotherapy. The patients were classified into three groups based on these ratios: group 1, ≥ 1.00; group 2, 0.85–0.99; group 3, < 0.85. The overall survival (OS) and surgical resection rates were estimated. Fifty-eight patients with LAPC who received first-line FOLFIRINOX were analyzed. Fifteen (25.9%) patients who underwent resection showed maintained BMI, SM, and AT as compared to the patients who did not undergo resection. As the SM ratio decreased, the risk for death increased significantly. Further, the resection rate was significantly higher in patients with maintained SM compared to those with low SM ratio. On the contrary, the change in AT ratio was not associated with OS and resection rate; however, significant decrease in AT more than 15% showed poor clinical outcomes. Maintenance of SM during chemotherapy is a reliable prognostic factor indicating longer OS and higher resection rate.

**Supplementary Information:**

The online version contains supplementary material available at 10.1186/s13104-021-05681-x.

## Introduction

Pancreatic cancer (PC) is the fourth leading cause of cancer-related deaths and has been projected to increase dramatically to become the second cause of cancer-related deaths by 2030 in the United States (U.S.) [1, 2]. Patients with cancer are at an increased risk for muscle loss via two distinct mechanisms: sarcopenia, defined as the muscle atrophy that occurs with aging or immobility, and cachexia, defined as cytokine-mediated degradation of muscle and adipose tissue. Both wasting disorders are prevalent; among patients with cancer, 15–50% are sarcopenic and 25–80% are cachectic [3]. Cancer cachexia, which is characterized by weight loss, muscle wasting, and fat tissue depletion is commonly observed in patients with PC [4]. The loss of skeletal muscle (SM) and adipose tissue (AT) has been shown to adversely affect the patient survival in many cancers [5–7]. It has also been reported that sarcopenia at presentation is associated with an unfavorable prognosis in patients with both resectable and advanced PC [8–10]. The aforementioned reports suggest that a series of anthropometric changes during chemotherapy may provide an important information for prognosis in patients with PC. In most studies, the estimates of body compositions have been derived from the cross-sectional data, and not from the longitudinal data. At least one third of patients are diagnosed with locally advanced PC (LAPC) with extensive vascular involvement. The treatment for patients with LAPC primarily involves systemic chemotherapy, such as gemcitabine plus nab-paclitaxel or FOLFIRINOX [11, 12]. Therefore, in the present study, we investigated the effect of the longitudinal changes in body composition during the first-line FOLFIRINOX chemotherapy on the prognosis of patients with LAPC.

## Main text

### Methods

#### Study population

Patients with LAPC who were treated with first-line FOLFIRINOX at Seoul National University Bundang Hospital (SNUBH) from April 2012 to June 2016 were enrolled. The current study was approved by the Institutional Review Board of SNUBH (IRB approval number: B-1807–481-102).

The inclusion criteria for patients in this study were: (1) pathologically proven adenocarcinoma; (2) clinically diagnosed with LAPC according to the International Study Group of Pancreatic Surgery criteria [13]; (3) received first-line FOLFIRINOX chemotherapy; (4) Eastern Cooperative Oncology Group (ECOG) performance score of 0 or 1; and (5) availability of two computed tomography (CT) scans (one at the time of diagnosis and another after 6 months of FOLFIRINOX chemotherapy). The following patients were excluded from the study: (1) patients who did not receive FOLFIRINOX chemotherapy; (2) patients with the recurrence of tumor; and (3) patients with poor performance status (ECOG ≥ 2).

#### Chemotherapy regimen/dose and follow-up

The FOLFIRINOX regimen was administered to the patients at the following doses: oxaliplatin, 85 mg/m^2^; irinotecan, 180 mg/m^2^; leucovorin, 400 mg/m^2^; and fluorouracil, 400 mg/m^2^ given as a bolus, followed by 2,400 mg/m^2^ given as a 46-h continuous infusion, every 2 weeks. Modification of the dose was determined by the clinician based on toxicity and patient preference. Treatment response was evaluated by CT scan after an average of three cycles of FOLFIRINOX chemotherapy using Response Evaluation Criteria in Solid Tumours (RECIST) criteria, version 1.1. The cumulative relative dose intensity (cRDI, %), which considered the modification of dose intensity as well as cycle duration, was calculated using the RDI calculator at http://www.hwang-lab.com.

#### Anthropometric measurements

**Fig. 1:**
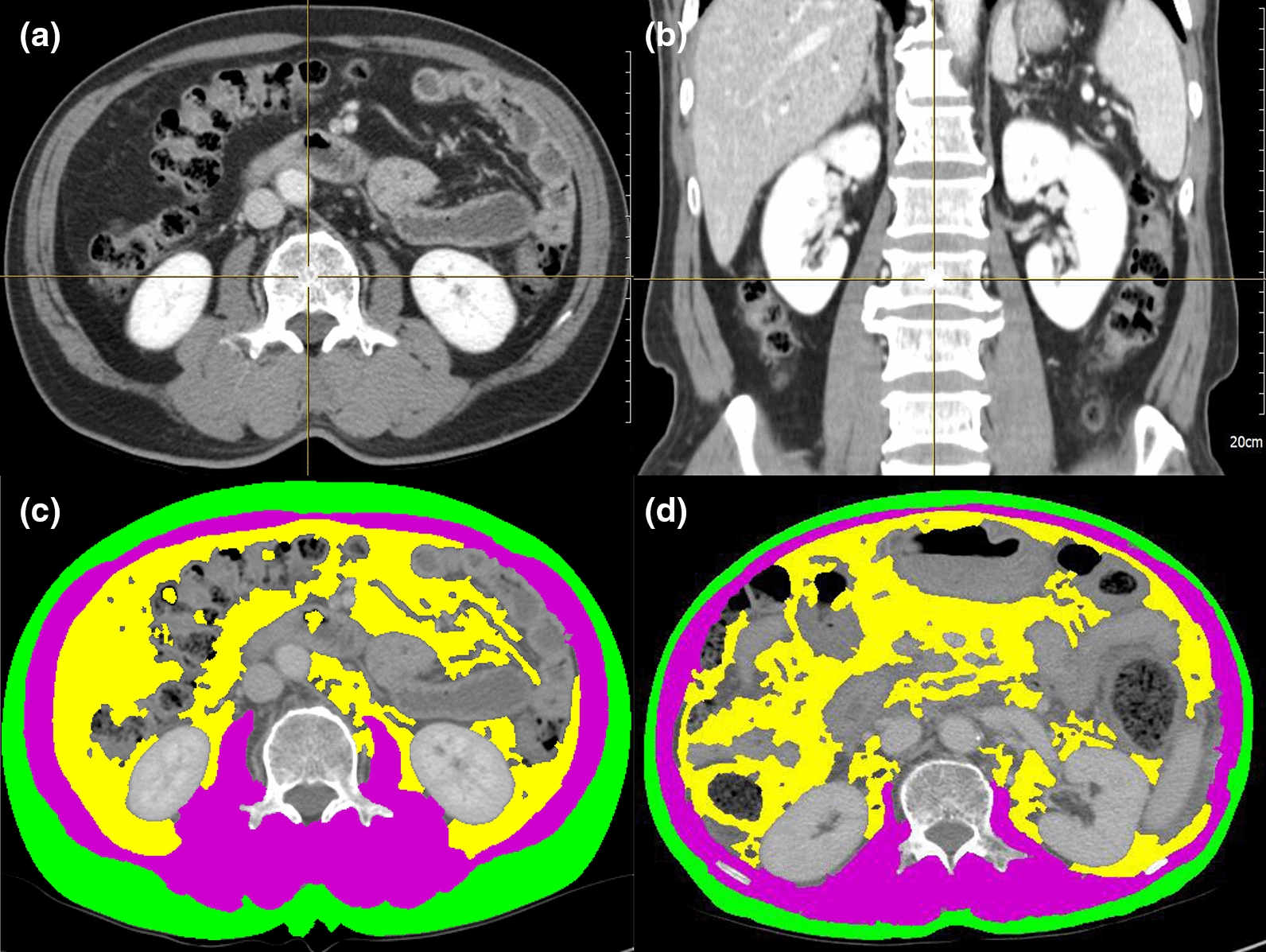
The quantification of body composition using computed tomography (CT) scan at the level of third lumbar vertebra (L3). **a**, **b** Cross-sectional image at L3 level that measured the skeletal muscle mass and fat mass. **c** CT image before FOLFIRINOX chemotherapy. **d** CT image after 6 months of FOLFIRINOX chemotherapy. The purple color indicates skeletal muscle, yellow indicates visceral adipose tissue, and green indicates subcutaneous adipose tissue

Initial anthropometric values, including height and body weight, were retrieved from the medical records, and were used to calculate the body mass index (BMI, kg/m^2^). Serial CT scans at the time of diagnosis and after 6 months were used to quantify body composition, including the SM and AT areas. The boundaries of the body composition area were drawn manually on the serial cross-sectional CT images at the level of third lumbar vertebra (L3) and measurements were recorded using SliceOmatic software (v5.0; Tomovision, Canada) (Fig. 1). The anthropometric data were used to calculate the ratios by dividing the post-chemotherapy values by the pre-chemotherapy values. SM and AT ratios were classified into three groups as follows: group 1, ≥ 1.00; group 2, 0.85–0.99; and group 3, < 0.85. The overall survival (OS), based on the SM and AT ratio groups, was estimated.

#### Statistical analyses

Categorical variables are presented as frequency and proportion, and continuous variables as mean ± standard deviation. Continuous variables with normal distribution were analyzed using the Student’s t-test. Differences among the groups were tested using the chi-square test, or paired t-test. Survival time was estimated using the Kaplan–Meier survival curves and compared using the log-rank test. Hazard ratios (HRs) and 95% confidence intervals (95% CIs) for the univariate model of OS were calculated using the Cox regression analysis. A two-sided *p*-value of < 0.05 was considered statistically significant. The statistical analyses were performed using the IBM SPSS, version 22 for Windows (IBM Inc., Armonk, NY, USA).

### Results

#### Patient characteristics

In the present study, initially 58 patients with LAPC were recruited from April 2012 to June 2016. The CT images before and after the administration of FOLFIRINOX chemotherapy were analyzed. After chemotherapy, 15 (25.9%) patients underwent surgical resection.

Table 1 summarizes the demographic characteristics of the participants. The median age was 62.1 years, and 34 (58.6%) patients were men. The median tumor size was 3.8 cm and the tumor was located in the head and/or neck of the pancreas in 39 (67.2%) patients. At first presentation, the anthropometric data were as follows (mean ± standard deviation): BMI, 21.7 ± 2.9 kg/m^2^; SM area, 111.8 ± 24.9 cm^2^; and AT area, 202.1 ± 85.6 cm^2^. All variables measured at the time of diagnosis showed no statistically significant differences between the resection group and the non-resection group.

**Table 1:** Baseline characteristics of the study population (n = 58)

Patient characteristics	Resection (n = 15)	Non-resection (n = 43)	Total (n = 58)	*p*-value
Age (years)	60.0 ± 10.5	62.8 ± 8.4	62.1 ± 9.0	0.292
Male, n (%)	6 (40.0%)	28 (65.1%)	34 (58.6%)	0.163
ECOG score, n (%)				0.531
0	8 (53.3%)	17 (39.5%)	25 (43.1%)	
1	7 (46.7%)	26 (60.5%)	33 (56.9%)	
DM, n (%)	6 (40.0%)	20 (46.5%)	26 (44.8%)	0.892
Tumor size (cm)	3.7 ± 0.7	3.9 ± 1.5	3.8 ± 1.3	0.434
Tumor location, n (%)				0.542
Head/neck	8 (53.3%)	31 (72.1%)	39 (67.2%)	
Body/tail	7(46.7%)	12 (27.9%)	19 (32.8%)	
Vessel invasion, n (%)				0.266
Artery	2 (13.3%)	9 (20.9%)	11 (19.0%)	
Vein	0 (0.0%)	5 (11.6%)	5 (8.6%)	
Both	13 (86.7%)	29 (67.4%)	42 (72.4%)	
CA 19–9 (U/mL)	576 ± 1,220	963 ± 1,833	863 ± 1,695	0.452
CEA (ng/mL)	9.2 ± 19.3	5.4 ± 10.1	6.4 ± 13.0	0.469
FOLFIRINOX cycles [median (range)]	11 (2–16)	8 (1–20)	9 (1–20)	0.193
≥ 6 cycles	14 (93.3%)	33 (76.7%)	47 (81.0%)	0.304
cRDI (%)	72.6 ± 12.4	66.4 ± 15.2	68.0 ± 14.7	0.164
≥ 70%	8 (53.3%)	21 (48.8%)	29 (50.0%)	1.000
BMI (kg/m^2^)	22.7 ± 2.6	21.3 ± 3.0	21.7 ± 2.9	0.110
SM area (cm^2^)	107.4 ± 26.6	113.3 ± 24.4	111.8 ± 24.9	0.435
AT area (cm^2^)	211.4 ± 66.3	198.8 ± 91.9	202.1 ± 85.6	0.627

The data are presented as mean ± standard deviation or n (%)

*ECOG* Eastern Cooperative Oncology Group, *DM* diabetes mellitus, *CA19-9* carbohydrate antigen 19–9, *CEA* carcinoembryonic antigen, *cRDI* corrected relative dose intensity, *BMI* body mass index, *SM* skeletal muscle, *AT* adipose tissue

The patients received an average of 9 (range, 1–20) cycles of FOLFIRINOX chemotherapy. The resection group received more cycles of FOLFIRINOX chemotherapy than the non-resection group, but the difference was not statistically significant (11 vs. 8 cycles; *p* = 0.193). The cRDI also did not show significant difference between the resection and non-resection groups (72.6% vs. 66.4%; *p* = 0.164).

#### Anthropometric changes during the FOLFIRINOX chemotherapy

As shown in Table 2, the body compositions changed after the FOLFIRINOX chemotherapy. During the 6 months of chemotherapy, BMI, SM area, and AT area decreased by 0.5 kg/m^2^, 12.6 cm^2^, and 24.1 cm^2^, respectively. Among the 15 (25.9%) patients who underwent surgery after chemotherapy, these three indicators were maintained or increased (BMI was the same; SM area increased by an average of 0.3 cm^2^; AT area increased by 3.2 cm^2^). However, these three anthropometric indices were significantly reduced in the unresected group (BMI decreased by 0.7 kg/m^2^; SM area decreased by 17.1 cm^2^; AT area decreased by 33.6 cm^2^).

**Table 2:** Changes of body mass index, skeletal muscle area, and adipose tissue area during the first-line FOLFIRINOX chemotherapy

Variables	Subgroups	Before chemotherapy	After chemotherapy	*p*-value	Difference (after-before)	Ratio (after/before)
BMI (kg/m^2^)	Resection (n = 15)	22.7 ± 2.6	22.7 ± 2.8	0.935	0.0 ± 2.2	1.013 ± 0.104
	No resection (n = 43)	21.3 ± 3.0	20.6 ± 3.2	0.013	− 0.7 ± 1.7	0.988 ± 0.078
	Total (n = 58)	21.7 ± 2.9	21.2 ± 3.2	0.052	− 0.5 ± 1.8	0.997 ± 0.086
SM area (cm^2^)	Resection (n = 15)	107.4 ± 26.6	107.8 ± 27.3	0.889	0.3 ± 9.2	1.006 ± 0.099
	No resection (n = 43)	113.3 ± 24.4	96.2 ± 22.9	< 0.001	− 17.1 ± 13.6	0.853 ± 0.116
	Total (n = 58)	111.8 ± 24.9	99.2 ± 24.4	< 0.001	− 12.6 ± 14.7	0.911 ± 0.130
AT area (cm^2^)	Resection (n = 15)	211.4 ± 66.3	214.6 ± 63.4	0.832	3.2 ± 57.0	1.062 ± 0.353
	No resection (n = 43)	198.8 ± 91.9	165.2 ± 83.4	< 0.001	− 33.6 ± 56.8	0.861 ± 0.253
	Total (n = 58)	202.1 ± 85.6	178.0 ± 81.2	0.003	− 24.1 ± 58.7	0.889 ± 0.292

The data are presented as mean ± standard deviation

*BMI* body mass index, *SM* skeletal muscle, *AT* adipose tissue

#### Effect of anthropometric changes on the clinical outcome

Additional file 1: Table S1 shows the effect of anthropometric changes during FOLFIRINOX chemotherapy on survival and resectability. The median OS of all 58 patients with LAPC was 16.1 months during a median duration of follow-up of 22.1 months. Regarding the SM ratio, 7 (12.1%), 31 (53.4%), and 20 (34.5%) patients were in group 1 (≥ 1.00), group 2 (0.85–0.99), and group 3 (< 0.80), respectively. The median OS (95% CI) based on the SM ratios was as follows: group 1, not reached; group 2, 19.1 (12.4–25.7) months; and group 3, 12.0 (9.0–15.0) months (Additional file 2: Figure S1a, Additional file 3: Figure S2a). As the SM ratio decreased, the risk of death increased significantly (group 1, reference; group 2, HR: 4.871, 95% CI: 1.136–20.882; group 3, HR: 11.212, 95% CI: 2.527–49.738; *p* < 0.001). The likelihood of resection was significantly higher in patients with maintained SM as compared to those with low SM ratio (group 1, 71.4%; group 2, 32.3%; group 3, 0%; *p* < 0.001).

Based on the AT ratio, there were 16 (27.6%), 20 (34.5%), and 22 (37.9%) patients in group 1 (≥ 1.00), group 2 (0.85–0.99), and group 3 (< 0.85), respectively. The median OS was not significantly different between group 1 (19.1 [11.5–26.7] months) and group 2 (17.5 [7.1–27.9] months) (HR: 1.282, 95% CI: 0.576–2.856) (Additional file 2: Figure S1b, Additional file 3: Figure S2b). However, if the AT ratio decreased by more than 15% (group 3), OS was significantly less (12.2 [11.1–13.3] months) (HR: 2.333, 95% CI: 1.067–5.103) than the group 1 (reference). Moreover, no statistically significant differences were observed in the resection rate among the different groups based on AT ratio (group 1, 31.3%; group 2, 35.0%; group 3, 13.6%; *p* = 0.243).

## Discussion

The present study showed the longitudinal changes in body composition and the associations of these changes with clinical outcomes in patients with LAPC during the first-line FOLFIRINOX chemotherapy. The principal findings of this study are: (i) the patients with well-maintained SM during chemotherapy were associated with higher resectability and longer OS than those with low SM ratio, even though cRDI was not different. (ii) The AT change during chemotherapy was not correlated with clinical outcomes; however, significant decrease in AT ratio showed poor clinical outcomes.

The sarcopenia at the time of diagnosis has been reported to be an independent prognostic factor of reduced survival in PC patients as well as in several cancers [5–10, 14–19], the underlying mechanism that links sarcopenia with poor survival has not been fully elucidated. A decrease in SM leads to aberrant energy homeostasis, impaired cell growth, insulin resistance, and immune dysfunction [20, 21]. In patients with PC, as well as nutritional deficiency, the insufficiency of pancreatic exocrine and endocrine functions, infection, and other metabolic factors can also contribute to the sarcopenia. The results of the present study showed that SM decrease during chemotherapy may be an important prognostic indicator for OS. SM decrease during chemotherapy may be a more important prognostic indicator than sarcopenia at the time of diagnosis. Many studies have reported that sarcopenic patients at the time of diagnosis have poor long-term outcomes [22–24]. The inability to maintain SM causes progressive functional impairment and a decreased quality of life, which in turn increases morbidity and mortality [25, 26].

Further, the findings of our study suggest that AT loss was not significantly associated with prognosis. The significant fat loss is associated with decreased survival in patients with PC, and this finding is consistent with the previously published data [27, 28]. Previous studies have shown that sarcopenia, sarcopenic obesity, and myosteatosis at the time of diagnosis are associated with decreased survival in patients with PC [29, 30]. However, owing to the complexity of PC, it is difficult to fully elucidate the underlying mechanisms associated with AT reduction and poor clinical outcomes.

Furthermore, a study has implicated tumor Fn14 (tumor necrosis factor receptor superfamily member 12A; TNFRSF12A) as an inducer of cachexia in mice models, and it was shown that the antibodies against Fn14 prevented tumor-induced cachexia and extended lifespan without chemotherapy [31]. This suggests that regardless of chemotherapy, survival time can be prolonged if cachexia and sarcopenia are prevented. The efforts to maintain SM, including dietary or physical rehabilitation programs, concurrent administration of nutritional supplements, and/or drugs targeting chronic inflammation and cachexia may represent a critical adjuvant strategy during chemotherapy [32].The determination of body compartments is an easy and quick tool that can be added without additional radiation exposure or cost. Anthropometric information can be easily applied in clinical setting with the potential to improve nutritional care and chemotherapy dose calculation.

## Conclusion

In conclusion, the anthropometric changes during the first-line FOLFIRINOX chemotherapy were associated with the clinical outcome in patients with LAPC. The findings of our study suggest that the patients with well-maintained SM during chemotherapy have longer OS and higher resectability.

## Limitations

The present study has some limitations. First, owing to its retrospective nature, we were unable to identify a causal relationship between sarcopenia and poor survival, and only revealed an association between them. Second, the information on standardized nutritional counseling during chemotherapy was missing. Despite these limitations, this study has several strengths. First, the longitudinal assessment of serial anthropometric measurements was used throughout the course of chemotherapy in patients with PC. Progressive loss of SM during chemotherapy was more closely related to shorter OS than pretreatment sarcopenia. Because, SM volume decreases over time with the tumor progression, it should ideally be evaluated longitudinally. Second, body composition can be evaluated by CT scan, which has the potential to become a feasible and clinically relevant measure.

## Supplementary Information

**Additional file 1: Table S1.** Median overall survival, resection rate, and chemotherapy dose according to anthropometric changes during FOLFIRINOX chemotherapy.

**Additional file 2: Figure S1.** Anthropometric changes during six months of FOLFIRINOX chemotherapy (a) changes in the skeletal muscle mass (b) changes in the total adipose tissue mass. The resection group is indicated by red color and non-resection group is indicated by gray color.

**Additional file 3: Figure S2.** Kaplan–Meier curves showing the relationship between anthropometric changes and overall survival (OS) (a) skeletal muscle (SM) ratio and OS (b) total adipose tissue (AT) ratio and OS.

## Data Availability

The datasets used and/or analyzed during the current study are available from the corresponding author on reasonable request.

## Abbreviations

PC: Pancreatic cancer
LAPC: Locally advanced pancreatic cancer
SNUBH: Seoul National University Bundang Hospital
CT: Computed tomography
RECIST: Response evaluation criteria in solid tumours
cRDI: Cumulative relative dose intensity
OS: Overall survival
ECOG: Eastern Cooperative Oncology Group
BMI: Body mass index
SM: Skeletal muscle
AT: Adipose tissue
HR: Hazard ratio
95% CI: 95% Confidence intervals
CA 19-9: Carbohydrate antigen 19-9
CEA: Carcinoembryonic antigen

## References

[1] Siegel RL, Miller KD, Fuchs HE, Jemal A (2021). Cancer statistics, 2021. CA Cancer J Clin.

[2] Rahib L, Smith BD, Aizenberg R, Rosenzweig AB, Fleshman JM, Matrisian LM (2014). Projecting cancer incidence and deaths to 2030: the unexpected burden of thyroid, liver, and pancreas cancers in the United States. Cancer Res.

[3] Peterson SJ, Mozer M (2017). Differentiating sarcopenia and cachexia among patients with cancer. Nutr Clin Pract.

[4] Ozola Zalite I, Zykus R, Francisco Gonzalez M, Saygili F, Pukitis A, Gaujoux S, Charnley RM, Lyadov V (2015). Influence of cachexia and sarcopenia on survival in pancreatic ductal adenocarcinoma: a systematic review. Pancreatology.

[5] Hamaguchi Y, Kaido T, Okumura S, Kobayashi A, Shirai H, Yao S, Yagi S, Kamo N, Seo S, Taura K (2019). Preoperative visceral adiposity and muscularity predict poor outcomes after hepatectomy for hepatocellular carcinoma. Liver Cancer.

[6] da Cunha LP, Silveira MN, Mendes MCS, Costa FO, Macedo LT, de Siqueira NS, Carvalheira JBC (2019). Sarcopenia as an independent prognostic factor in patients with metastatic colorectal cancer: A retrospective evaluation. Clin Nutr ESPEN.

[7] Rossi F, Valdora F, Bignotti B, Torri L, Succio G, Tagliafico AS (2019). Evaluation of body Computed Tomography-determined sarcopenia in breast cancer patients and clinical outcomes: a systematic review. Cancer Treat Res Commun.

[8] Sugimoto M, Farnell MB, Nagorney DM, Kendrick ML, Truty MJ, Smoot RL, Chari ST, Moynagh MR, Petersen GM, Carter RE (2018). Decreased skeletal muscle volume is a predictive factor for poorer survival in patients undergoing surgical resection for pancreatic ductal adenocarcinoma. J Gastrointest Surg.

[9] Choi Y, Oh DY, Kim TY, Lee KH, Han SW, Im SA, Kim TY, Bang YJ (2015). Skeletal muscle depletion predicts the prognosis of patients with advanced pancreatic cancer undergoing palliative chemotherapy, independent of body mass index. PLoS ONE.

[10] Park I, Choi SJ, Kim YS, Ahn HK, Hong J, Sym SJ, Park J, Cho EK, Lee JH, Shin YJ (2016). Prognostic factors for risk stratification of patients with recurrent or metastatic pancreatic adenocarcinoma who were treated with gemcitabine-based chemotherapy. Cancer Res Treat.

[11] Philip PA, Lacy J, Portales F, Sobrero A, Pazo-Cid R, Manzano Mozo JL, Kim EJ, Dowden S, Zakari A, Borg C (2020). Nab-paclitaxel plus gemcitabine in patients with locally advanced pancreatic cancer (LAPACT): a multicentre, open-label phase 2 study. Lancet Gastroenterol Hepatol.

[12] Marthey L, Sa-Cunha A, Blanc JF, Gauthier M, Cueff A, Francois E, Trouilloud I, Malka D, Bachet JB, Coriat R (2015). FOLFIRINOX for locally advanced pancreatic adenocarcinoma: results of an AGEO multicenter prospective observational cohort. Ann Surg Oncol.

[13] Bockhorn M, Uzunoglu FG, Adham M, Imrie C, Milicevic M, Sandberg AA, Asbun HJ, Bassi C, Buchler M, Charnley RM (2014). Borderline resectable pancreatic cancer: a consensus statement by the International Study Group of Pancreatic Surgery (ISGPS). Surgery.

[14] Daly LE, Ni Bhuachalla EB, Power DG, Cushen SJ, James K, Ryan AM (2018). Loss of skeletal muscle during systemic chemotherapy is prognostic of poor survival in patients with foregut cancer. J Cachexia Sarcopenia Muscle.

[15] Sandini M, Patino M, Ferrone CR, Alvarez-Perez CA, Honselmann KC, Paiella S, Catania M, Riva L, Tedesco G, Casolino R (2018). Association between changes in body composition and neoadjuvant treatment for pancreatic cancer. JAMA Surg.

[16] El Amrani M, Vermersch M, Fulbert M, Prodeau M, Lecolle K, Hebbar M, Ernst O, Pruvot FR, Truant S (2018). Impact of sarcopenia on outcomes of patients undergoing pancreatectomy: a retrospective analysis of 107 patients. Medicine (Baltimore).

[17] Choi MH, Yoon SB, Lee K, Song M, Lee IS, Lee MA, Hong TH, Choi MG (2018). Preoperative sarcopenia and post-operative accelerated muscle loss negatively impact survival after resection of pancreatic cancer. J Cachexia Sarcopenia Muscle.

[18] Dalal S, Hui D, Bidaut L, Lem K, Del Fabbro E, Crane C, Reyes-Gibby CC, Bedi D, Bruera E (2012). Relationships among body mass index, longitudinal body composition alterations, and survival in patients with locally advanced pancreatic cancer receiving chemoradiation: a pilot study. J Pain Symptom Manage.

[19] Basile D, Parnofiello A, Vitale MG, Cortiula F, Gerratana L, Fanotto V, Lisanti C, Pelizzari G, Ongaro E, Bartoletti M (2019). The IMPACT study: early loss of skeletal muscle mass in advanced pancreatic cancer patients. J Cachexia Sarcopenia Muscle.

[20] Biolo G, Cederholm T, Muscaritoli M (2014). Muscle contractile and metabolic dysfunction is a common feature of sarcopenia of aging and chronic diseases: from sarcopenic obesity to cachexia. Clin Nutr.

[21] Demontis F, Piccirillo R, Goldberg AL, Perrimon N (2013). The influence of skeletal muscle on systemic aging and lifespan. Aging Cell.

[22] Prado CM, Lieffers JR, McCargar LJ, Reiman T, Sawyer MB, Martin L, Baracos VE (2008). Prevalence and clinical implications of sarcopenic obesity in patients with solid tumours of the respiratory and gastrointestinal tracts: a population-based study. Lancet Oncol.

[23] Okumura S, Kaido T, Hamaguchi Y, Kobayashi A, Shirai H, Yao S, Yagi S, Kamo N, Hatano E, Okajima H (2017). Visceral adiposity and sarcopenic visceral obesity are associated with poor prognosis after resection of pancreatic cancer. Ann Surg Oncol.

[24] Mintziras I, Miligkos M, Wachter S, Manoharan J, Maurer E, Bartsch DK (2018). Sarcopenia and sarcopenic obesity are significantly associated with poorer overall survival in patients with pancreatic cancer: Systematic review and meta-analysis. Int J Surg.

[25] Holly EA, Chaliha I, Bracci PM, Gautam M (2004). Signs and symptoms of pancreatic cancer: a population-based case-control study in the San Francisco Bay area. Clin Gastroenterol Hepatol.

[26] Arthur ST, Noone JM, Van Doren BA, Roy D, Blanchette CM (2014). One-year prevalence, comorbidities and cost of cachexia-related inpatient admissions in the USA. Drugs Context.

[27] Cooper AB, Slack R, Fogelman D, Holmes HM, Petzel M, Parker N, Balachandran A, Garg N, Ngo-Huang A, Varadhachary G (2015). Characterization of anthropometric changes that occur during neoadjuvant therapy for potentially resectable pancreatic cancer. Ann Surg Oncol.

[28] Di Sebastiano KM, Yang L, Zbuk K, Wong RK, Chow T, Koff D, Moran GR, Mourtzakis M (2013). Accelerated muscle and adipose tissue loss may predict survival in pancreatic cancer patients: the relationship with diabetes and anaemia. Br J Nutr.

[29] Kays JK, Shahda S, Stanley M, Bell TM, O'Neill BH, Kohli MD, Couch ME, Koniaris LG, Zimmers TA (2018). Three cachexia phenotypes and the impact of fat-only loss on survival in FOLFIRINOX therapy for pancreatic cancer. J Cachexia Sarcopenia Muscle.

[30] Tan BH, Birdsell LA, Martin L, Baracos VE, Fearon KC (2009). Sarcopenia in an overweight or obese patient is an adverse prognostic factor in pancreatic cancer. Clin Cancer Res.

[31] Johnston AJ, Murphy KT, Jenkinson L, Laine D, Emmrich K, Faou P, Weston R, Jayatilleke KM, Schloegel J, Talbo G (2015). Targeting of Fn14 prevents cancer-induced cachexia and prolongs survival. Cell.

[32] Wakabayashi H, Sakuma K (2014). Rehabilitation nutrition for sarcopenia with disability: a combination of both rehabilitation and nutrition care management. J Cachexia Sarcopenia Muscle.

