# Supplementary material for: Maintenance of skeletal muscle mass during FOLFIRINOX is a favorable prognostic factor in pancreatic cancer patients
Source: BMC Res Notes. 2021 Jul 15;14:272. doi: 10.1186/s13104-021-05681-x (PMC8281692; doi:10.1186/s13104-021-05681-x)
Supplement: Supplementary file 1 — Additional file 1: Table S1. Median overall survival, resection rate, and chemotherapy dose according to anthropometric changes during FOLFIRINOX chemotherapy. [file 13104_2021_5681_MOESM1_ESM.docx]

**Additional file 1: Table S1. Median overall survival, resection rate, and chemotherapy dose according to anthropometric changes during FOLFIRINOX chemotherapy**

|  | **n (%)** | **OS (months)**  **[median (range)]** | **HR (95% CI)** | ***p*-value** | **Resection** | ***p*-value** | **CTx. cycles** | ***p*-value** | **cRDI (%)** | ***p*-value** |
| --- | --- | --- | --- | --- | --- | --- | --- | --- | --- | --- |
| **Total** |  | 16.1 (13.1-19.1) | - | - | 15 (25.9%) |  | 8.9 ± 4.0 |  | 68.0 ± 14.7 |  |
| **SM ratio (after/before CTx.)** |  |  |  | <0.001 |  | <0.001 |  | 0.536 |  | 0.481 |
| **Group 1 (≥1.00)** | 7 (12.1%) | not reached | 1 (reference) |  | 5 (71.4%) |  | 10.0 ± 4.4 |  | 71.7 ± 15.2 |  |
| **Group 2 (0.85-0.99)** | 31 (53.4%) | 19.1 (12.4-25.7) | 4.871 (1.136-20.882) |  | 10 (32.3%) |  | 10.0 ± 4.0 |  | 70.4 ± 15.7 |  |
| **Group 3 (<0.85)** | 20 (34.5%) | 12.0 (9.0-15.0) | 11.212 (2.527-49.738) |  | 0 (0.0%) |  | 8.0 ± 3.6 |  | 67.2 ± 13.4 |  |
| **AT ratio (after/before CTx.)** |  |  |  | 0.077 |  | 0.243 |  | 0.131 |  | 0.426 |
| **Group 1 (≥1.00)** | 16 (27.6%) | 19.1 (11.5-26.7) | 1 (reference) |  | 5 (31.3%) |  | 11.0 ± 4.0 |  | 64.2 ± 10.9 |  |
| **Group 2 (0.85-0.99)** | 20 (34.5%) | 17.5 (7.1-27.9) | 1.282 (0.576-2.856) |  | 7 (35.0%) |  | 9.0 ± 2.9 |  | 68.9 ± 10.9 |  |
| **Group 3 (<0.85)** | 22 (37.9%) | 12.2 (11.1-13.3) | 2.333 (1.067-5.103) |  | 3 (13.6%) |  | 8.5 ± 4.7 |  | 71.4 ± 16.1 |  |

The data are presented as mean ± standard deviation or n (%).

Abbreviations: OS, overall survival; HR, hazard ratio; 95% CI, 95% confidence interval; CTx., chemotherapy; SM, skeletal muscle; AT, adipose tissue; cRDI, cumulative relative dose intensity
